# Net Carbon Emissions from Deforestation in Bolivia during 1990-2000 and 2000-2010: Results from a Carbon Bookkeeping Model

**DOI:** 10.1371/journal.pone.0151241

**Published:** 2016-03-18

**Authors:** Lykke E. Andersen, Anna Sophia Doyle, Susana del Granado, Juan Carlos Ledezma, Agnes Medinaceli, Montserrat Valdivia, Diana Weinhold

**Affiliations:** 1 Center for Environmental-Economic Modeling and Analysis (CEEMA), Institute for Advanced Development Studies (INESAD), La Paz, Bolivia; and Universidad Privada Boliviana, La Paz, Bolivia; 2 Center for Environmental-Economic Modeling and Analysis (CEEMA), Institute for Advanced Development Studies (INESAD), La Paz, Bolivia; 3 Conservation International–Bolivia, La Paz, Bolivia; 4 London School of Economics and Political Science, London, United Kingdom; Shandong University, CHINA

## Abstract

Accurate estimates of global carbon emissions are critical for understanding global warming. This paper estimates net carbon emissions from land use change in Bolivia during the periods 1990–2000 and 2000–2010 using a model that takes into account deforestation, forest degradation, forest regrowth, gradual carbon decomposition and accumulation, as well as heterogeneity in both above ground and below ground carbon contents at the 10 by 10 km grid level. The approach permits detailed maps of net emissions by region and type of land cover. We estimate that net CO_2_ emissions from land use change in Bolivia increased from about 65 million tons per year during 1990–2000 to about 93 million tons per year during 2000–2010, while CO_2_ emissions per capita and per unit of GDP have remained fairly stable over the sample period. If we allow for estimated biomass increases in mature forests, net CO_2_ emissions drop to close to zero. Finally, we find these results are robust to alternative methods of calculating emissions.

## Introduction

With more than 51 million hectares of forest [1], Bolivia is home to the 7^th^ largest area of tropical rainforest [2] and is among the dozen countries with highest terrestrial biodiversity [3]. However, deforestation rates have increased rapidly during the last three decades, and during the period from 2000–2010, about 430,000 hectares of forest were lost annually [4], primarily due to the expansion of the agricultural frontier [5]. Deforestation on this scale causes CO_2_ emissions of about one hundred million tons per year, making deforestation responsible for more than 80% of Bolivia’s total CO_2_ emissions [6].

However, even as deforestation progresses apace, both satellite images and ground observations have shown that a significant and increasing area of previously deforested land is currently experiencing a process of natural forest regeneration [4], absorbing CO_2_ from the atmosphere. For example, SERNAP [4] identifies almost 1.5 million hectares of regenerating forest in 2010, implying that about 23% of the 6.5 million hectares that were deforested over the last 3 decades may be currently in the process of natural regeneration.

Traditionally, most carbon accounting approaches have assumed that forests are either intact or cleared, and that once cleared, stay cleared forever. The purpose of this paper is to take into account the fact that forests are found in a wide variety of states (mature, degraded, cleared, or in various states of natural regeneration) to more accurately estimate the net carbon emissions in Bolivia during the 1990s and 2000s. We adopt a dynamic modelling approach that uses highly disaggregated national data and which also takes into account changes in below-ground carbon stocks (here defined as carbon in live roots and organic soil carbon in the upper 100 cm of soils). This pixel-level carbon bookkeeping approach has been labelled by the Intergovernmental Panel on Climate Change (IPCC) a “Tier 3” methodology—the most detailed and accurate method, although also the most data demanding [7].

We also compare our “Tier 3” estimates to those from simpler "Tier 1" carbon calculations based only on average national deforestation rates and average aboveground carbon densities for all Bolivian forests (e.g. [8], [9]). We find our Tier 3 estimates are less than 10% lower than the much-easier-to-compute Tier 1 estimates, suggesting that overall total estimates based on less data, using the simpler approaches are likely to be quite reliable.

The remainder of the paper is organised as follows: Section 2 describes the main land use change patterns observed in Bolivia during the period 1990 to 2010, according to satellite images from NASA Geocover for 1990 and 2000 and from Landsat 5 TM for 2010. Section 3 outlines the carbon accounting methodology and section 4 discusses the results and explores the spatial and temporal distribution of carbon emissions. Section 5 presents a sensitivity analysis, and section 6 concludes.

## Forest Cover and Land Use Change in Bolivia

Most land use change in Bolivia follows several typical patterns common in land-abundant tropical countries (e.g. [10]) and can be thought of as variations on typical slash-and-burn agriculture that progresses through some or all of four phases: (i) land clearing and forest burning (often at an industrial scale); (ii) agropastoral use of land; (iii) soil exhaustion and/or weed infestation, land abandonment; and (iv) the regrowth of secondary vegetation. In some cases phase (iv) is long fallow, with agriculture returning once the soil has sufficiently recovered, while in other cases the land may be completely abandoned by the owner. Where land values are high enough to induce land use intensification (such as near urban agglomerations), phase (iii) can be forestalled or eliminated with the use of fertilizers, pesticides, and/or innovative farm management practices, allowing land to remain in agricultural use indefinitely ([11], [12]). In other circumstances forests may be cleared but forgoing phase (ii) of agricultural use. This pattern has been observed in Brazil where settlers clear land simply to establish property rights [13], while in Bolivia such a transition is more generally due to accidental fire [14]. When the slash-and-burning takes place on an industrial-scale level, such as in the case of soy bean cultivation, regeneration may be more difficult even if the land is eventually abandoned. In addition the new vegetation may have a very different species composition than the original forest. Finally, we also observe situations where forests are not completely cleared but instead partly degraded, usually from selective logging or accidental forest fires. Thus, we observe forests in many different states, with different levels of carbon storage, and to our knowledge this is the first carbon accounting study to take this diversity of land use outcomes into account.

In order to generate estimates of the carbon emissions associated with these land use changes, the carbon content of both the forests, soils, and agricultural land must be estimated, as must the carbon transition curves for each of the relevant land use changes. We begin by generating a 10 by 10 km grid of the entire territorial area of Bolivia. Each grid square, or pixel, is then populated with data on vegetation cover and carbon content for the years 1990, 2000, and 2010 (assigning just one predominant forest type to each pixel-year to keep the calculations tractable) by combining data from two recently released data sets on forest cover in Bolivia with two global maps on aboveground living biomass and organic carbon contents in soils. Specifically, vegetation cover and forest data come from the Bolivian National Service of Protected Areas (SERNAP), [4], which analysed satellite images from NASA Geocover for 1990 and 2000 and from Landsat 5 TM for 2010, as well as from the 2013 Forest Map published by the Ministry of Environment and Water—Amazon Cooperation Treaty (MMAyA-OTCA) [1], which divides all Bolivian forests into nine categories. Living aboveground biomass data for Bolivia is extracted from maps developed by the Woods Hole Research Institute [15], and total organic carbon content in soils (for the top 100 cm) comes from the Harmonized World Soil Database developed by FAO et al. [16]. To date no one has mapped carbon contents in tree roots in Bolivia, so for this component of belowground biomass we use IPCC default ratios for below ground to above ground biomass in different types of forests [7]. The Supporting Information files provide detailed specifics about each dataset and how the corresponding values of vegetation cover and carbon content were extracted and estimated.

Table A in the S1 Supporting Information file catalogues the distribution of forest types in 2013 from MMAyA-OTCA [1]. Amazon forest is clearly the most important forest type, accounting for more than a third of all Bolivian forests. This is followed by Chaco forest, Chiquitano forest and Yungas forest, each accounting for close to a sixth of the total forest cover. These are followed in importance by the flooded forest of Beni and the Pantanal forest of Santa Cruz. Dry Inter-Andean forest and Andean forest together account for only 0.3% of all Bolivian forests.

Table B in the S2 Supporting Information file shows the land-use transitions experienced by all lands in Bolivia (excluding lakes and salt flats that do not belong to any municipality) between 1990 and 2000 and between 2000 and 2010 according to the SERNAP [4] dataset. The total amount of forest cover has decreased from 56.8 million hectares in 1990 to 55.0 million hectares in 2000 and to 52.6 million hectares in 2010. This implies a reduction of 4.2 million hectares of forest over 20 years, corresponding to an average forest loss of about 210.000 hectares per year or about 0.4% per year. At the same time, agriculture on previously forested land has increased from 1.9 million hectares in 1990 to 3.3 million hectares in 2000 and to 5.2 million hectares in 2010, for a total increase of 3.3 million hectares.

From Table B we can see that the difference between the 4.2 million hectare loss of forest cover and the 3.3 million hectare increase in land is largely explained by forest regrowth (e.g. transitions from agricultural area to regrowth). Several other types of transitions are also worth noting; for example, 0.8 million hectares transitioned directly from forest in 2000 to regrowth in 2010. This could happen, for example, if the area was cleared for agriculture in 2001 or 2002 and only used for a few years, and thus visibly in the process of regeneration by 2010. However, it could also happen due to forest degradation caused by selective logging or wildfires, giving the area the appearance (to a satellite) of young regenerating forest. We also observe that 1.27 million hectares transitioned directly from forest in 2000 to another kind of vegetation in 2010. This could, for instance, have been caused by extensive wildfires that turned the forest into something that now looks (to a satellite) like savannah instead of a regenerating forest.

Indeed, there is an inherent uncertainty in satellite derived measures of deforestation, as natural vegetation spans a dynamic continuum of densities, and the type of satellite images used also changes between 2000 and 2010. For example, Table B shows that an area of 1.33 million hectares seems to have changed from other vegetation in 2000 directly to something that looked like forest in 2010, which is difficult to explain. Fortunately, these odd transitions seem to approximately cancel each other out at the aggregate level.

## A "Tier 3" Carbon Bookkeeping Model

In order to estimate carbon emissions from land use change we employ a carbon bookkeeping model originally proposed by Houghton et al. [17] that is common in the literature (e.g. [15], [18], [19]) and involves tracking both the carbon released to the atmosphere from forests converted to agricultural land or degraded due to logging or forest fires, as well as the carbon accumulated as forests regenerate after abandonment. The model has two important parts: a set of equilibrium values for the carbon contents in different types of forests and in different types of land uses, and a set of response (transition) functions that indicate how carbon is decomposed and accumulated after changes in land use. This model allows a much more detailed analysis of how much carbon is stored at any point in time, and is therefore different from a carbon life-cycle analysis, which usually makes the simplifying assumption that all carbon is emitted immediately after land use change, and that the change is permanent.

### Above- and below- ground carbon estimates

**Fig 1:**
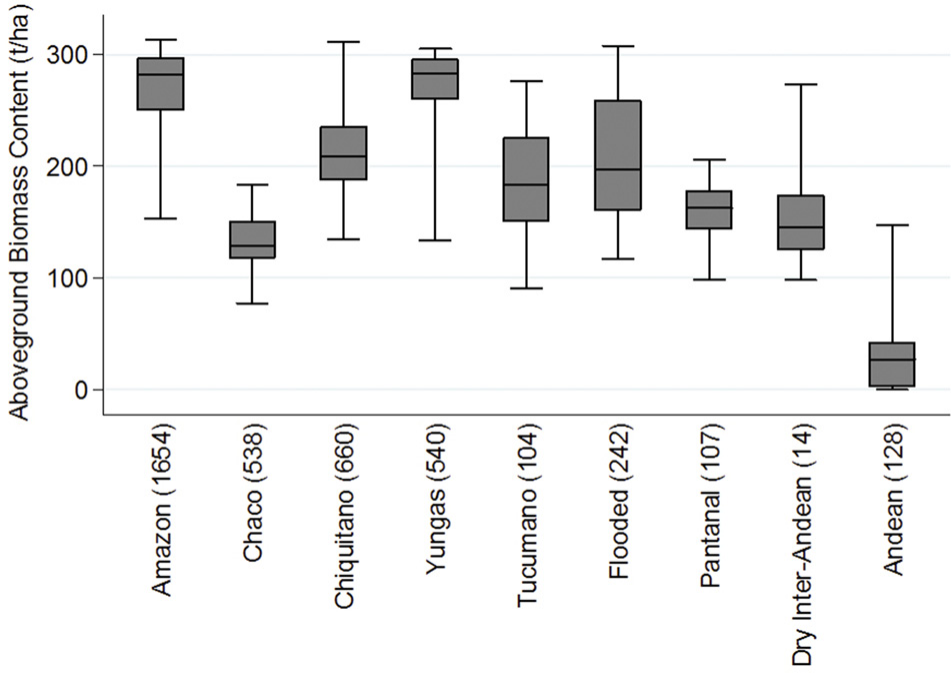
Distribution of aboveground biomass in virtually intact forests, by forest type. Source: Authors’ elaboration based on aboveground biomass measured by Baccini et al [15]. The outer bars show the 95% range of the biomass data while the box with the line in the middle show the 25^th^, 50^th^, and 75^th^ percentiles of the biomass data. The number in parenthesis after the forest type name indicates how many nearly intact pixels were used to calculate the biomass percentiles.

First, in order to calculate the typical carbon contents in different types of mature forests, we identify pixels with nearly intact forest as a reference for each of the forest types. We then find the corresponding above ground biomass from maps developed by the Woods Hole Research Institute, which uses a combination of co-located field measurements, LiDAR observations and imagery recorded from the Moderate Resolution Imaging Spectroradiometer (MODIS) [15]. Fig 1 shows the variation in average above ground biomass contents for pixels with nearly intact forest, for each of the nine different forest types. We then follow IPCC [7] and assume that roughly half of living biomass is carbon. Average above ground carbon contents (weighted by the forest shares presented in Table A of the S1 Supporting Information file) is 114 tC/ha.

Fig 1 also illustrates the substantial variation in biomass content around the median, even in intact ecosystems. This variation could complicate estimates of carbon emissions, as deforestation is not necessarily randomly distributed within each vegetation type [20]. In the Brazilian Amazon, for example, it has been shown that the areas that have been deforested so far are less dense than average Brazilian Amazon forest ([11]; [21]; [22]). If the same is true in Bolivia, using the median biomass for each forest type may lead to an overestimation of carbon emissions. In order to see how sensitive our results are to this possible bias, in section 5 below we repeat the analysis using the 25^th^ percentiles instead of the 50^th^ percentile for each forest type (see Table C in the S3 Supporting Information file for the relevant percentile values).

**Fig 2:**
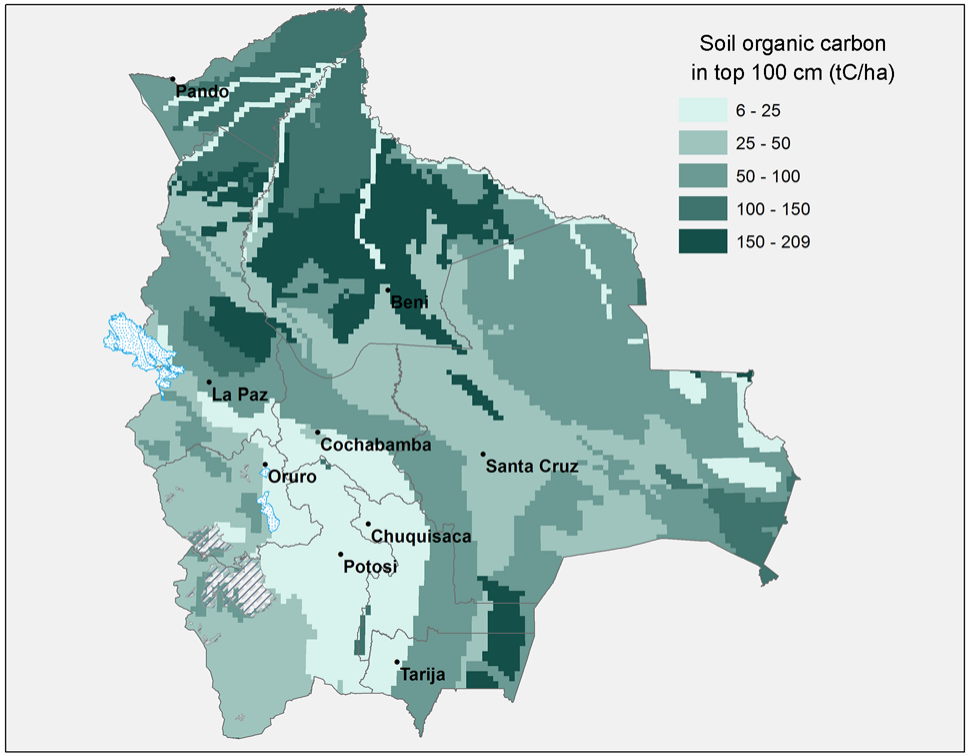
Map of Soil Organic Carbon density in Bolivia. Source: Authors’ elaboration based on data from the Harmonized World Soil Database, FAO et al. [16]

In addition to carbon in woody biomass we also calculate total carbon contents in soils (for the top 100 cm) by projecting data from the Harmonized World Soil Database developed by FAO et al. [16] on to our 10 by 10 km grid of Bolivia. All of these organic carbon observations were extracted for topsoil and subsoil for the dominant soil groups and units on the territory, except for Leptosols, the only soil group that lacks information for subsoil. This soil type is located mostly in the Andean, Dry Inter-Andean and Tucumano Forests (see Fig 2). The results of this exercise are illustrated in Fig 2, which shows that the most carbon rich soils are found in the flooded forests and prairies of Beni, the Yungas forests, and in the Chaco forests.

No maps of carbon contents in roots exist for Bolivia, nor are there sufficient field samples available to create one, so we use IPCC’s default ratios for belowground biomass to aboveground biomass for each forest type. Table D in the S4 Supporting Information file shows the parameters used.

### Carbon decomposition and regeneration curves

Since most forest is cleared by burning to make room for agriculture, we will assume that above- ground carbon contents immediately drop to the level found in crop land or pasture (5 tC/ha according to IPCC [7]). However, carbon initially held below ground is released to the atmosphere gradually and evidence suggests that soil carbon is quite resilient to forest disturbance above ground. For example, Berenguer et al.'s seminal 2014 study [23] on the effects of human-induced forest disturbances on soil carbon in the Brazilian Amazon finds that while above ground carbon differs substantially between undisturbed and disturbed forests, this is not the case with soil carbon, which seems very similar across all types of forests; if anything, soil carbon stocks actually seem to be slightly higher in disturbed forests. Likewise, a meta-analysis carried out by Guo and Gifford [24] finds that, on average, the change from forest to pasture caused an 8% *increase* in organic soil carbon. Thus, we assume that only if forests are converted to agriculture will carbon be emitted from soils, and following Houghton and Hackler [25], who review experimental evidence from South and Central America, we further assume that 25% of soil organic carbon in the top 100 cm is lost within 20 years. We also assume a linear below ground carbon decomposition curve from year 0 to year 20 of agricultural land with no further change.

**Fig 3:**
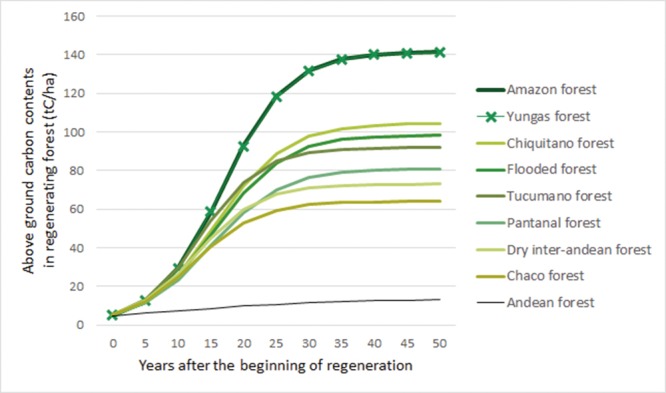
Above ground carbon regeneration curves, by forest type. Source: Authors’ elaboration based on parameters listed in Table E of the S5 Supporting Information file.

Following Reis and Andersen [26] we specify logistic carbon regeneration curves for above ground biomass, as this functional form allows rapid carbon accumulation in the beginning, when fast-growing pioneer species populate the area, and slowing convergence towards the original carbon contents in mature forests. Above ground carbon contents in regenerating forest is thus given by Eq (1):

$${CAR}_{v,a}=\frac{{CAP}_{v}}{1+e^{\left(\alpha_{v}-\beta_{v}a\right)}}$$

where *CAR*_*v*,*a*_ is above ground carbon contents in regrowth of age *a* for forest type *v*, *CAP*_*v*_ is the above ground carbon content in mature forest type *v*, and α_*v*_ and β_*v*_ are parameters that determine the exact shape of the logistic function. As discussed above, and following IPCC guidelines [7], we assume that the carbon content of the initial agricultural land is 5 tC/ha for all forest types, and following Houghton and Hackler [25], we further assume that humid tropical forest in Bolivia takes 40 years to regenerate to 99% of their maximum carbon content, while dryer and more seasonal forests take 35 years to recover. This leads to the aboveground carbon regeneration curves illustrated in Fig 3. Table E in the S5 Supporting Information file shows the corresponding parameters.

Following Houghton and Hackler [25] we assume that it takes twice as long for belowground carbon to regenerate as it took it to decompose, i.e. it will require 40 years for belowground carbon to return to its original level if it has been under agriculture for 20 years. This latter assumption is also consistent with Li et al. [27], which finds that significant soil carbon increases are only found 30 to 50 years after afforestation. Thus, we assume a linear belowground carbon regeneration curve with half the slope of the belowground carbon decomposition curve.

### Carbon contents in degraded forests

In satellite images, we sometimes observe plots that are neither mature forests, nor completely deforested, and these are classified as either degraded forest or as regenerating forest, depending on the history of the plot. If the plot has not previously been cleared, we assume it is degraded forest, usually due to selective logging, wildfires or natural factors such as storms, landslides, or river course changes. In our central estimates, we assume that degraded forest has the same aboveground carbon content as 25-year old regrowth (which has 85% of the carbon content of mature forest). This assumption is somewhat arbitrary, and carbon from some types of degradation may be released at different time scales (for example carbon stored in furniture or flooring made from selectively logged forests, or carbon emitted slowly over time from a river course change) potentially causing small over- or under-estimates. Thus we also conduct a sensitivity analysis where we assume much more severe degradation (carbon contents that are only 41% of those in mature forests). Finally, considering the strong field evidence of Berenguer et al. [23] from neighbouring Brazil, we assume that belowground carbon remains intact in degraded forests.

## Results

Overall our results suggest that during the last 20 years, Bolivia’s net CO_2_ emissions from land use change amounted to about 1.6 billion tons. Table 1 presents the aggregate estimates for each decade. Total emissions were 42% higher in 2000–2010 compared to 1990–2000, but once population growth is taken into account we find that *per capita* emissions from land use change increased only by about 12%, remaining close to 10 tCO_2_/person/year. Further taking into account GDP growth, we find that emissions per unit of GDP generated has actually *fallen* very slightly (by 0.04 kg/Bs.) between 1990–2000 and 2000–2010.

**Table 1:** Central estimates of net CO_2_ emissions from land use change in Bolivia, 1990–2000 and 2000–2010.

	1990–2000	2000–2010
Total CO_2_ emissions (tCO_2_)	653,858,680	926,094,559
Average annual CO_2_ emissions (tCO_2_/year)	65,385,868	92,609,456
Mid-period population	6,987,201	8,873,150
Average annual per capita CO_2_ emissions (tCO_2_/person/year)	9.4	10.4
Mid-period GDP (thousands of real Bolivianos of 1990)	18,626,778	26,657,738
Emissions per unit of GDP (kg/1990-Bs.)	3.5	3.5

Source: Authors’ estimations.

Note: GDP is measured in real (inflation-adjusted) Bolivianos of 1990 (1990-Bs.) so as to be comparable over time.

**Fig 4:**
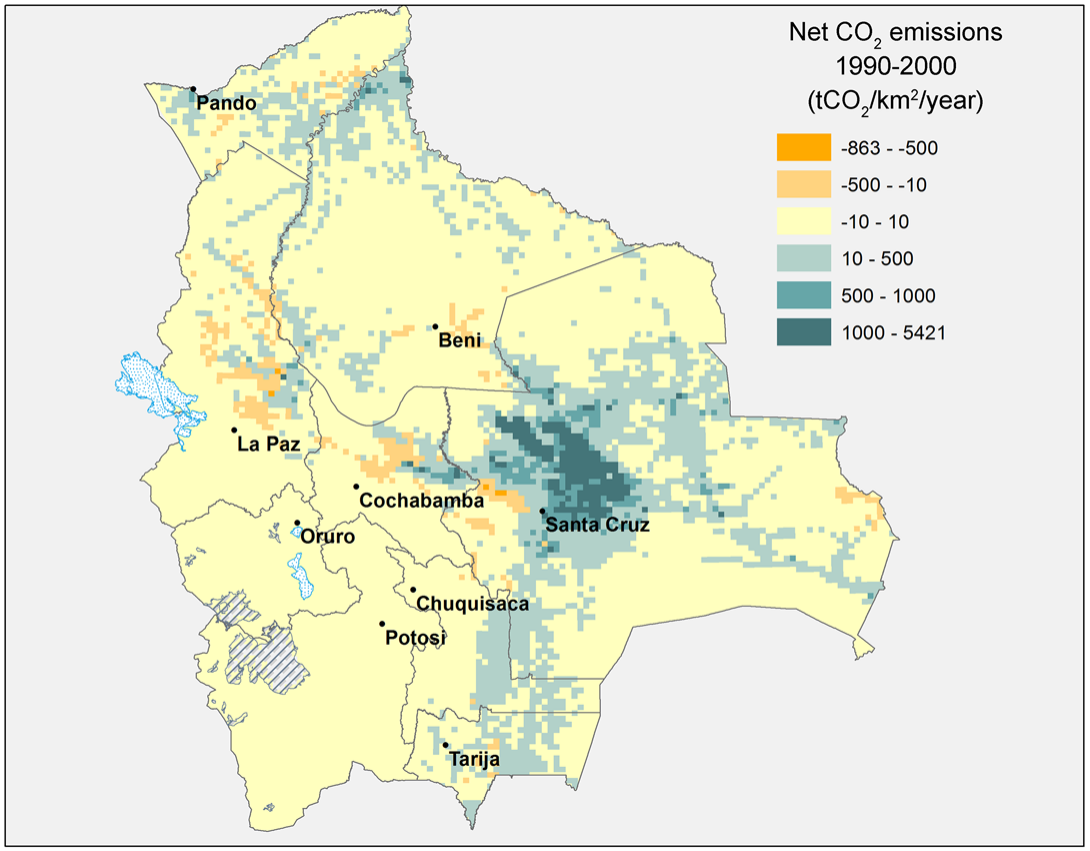
Annual net CO_2_ emissions from land use change, 1990–2000 (tCO_2_/km^2^/year). Source: Authors’ elaboration.

**Fig 5:**
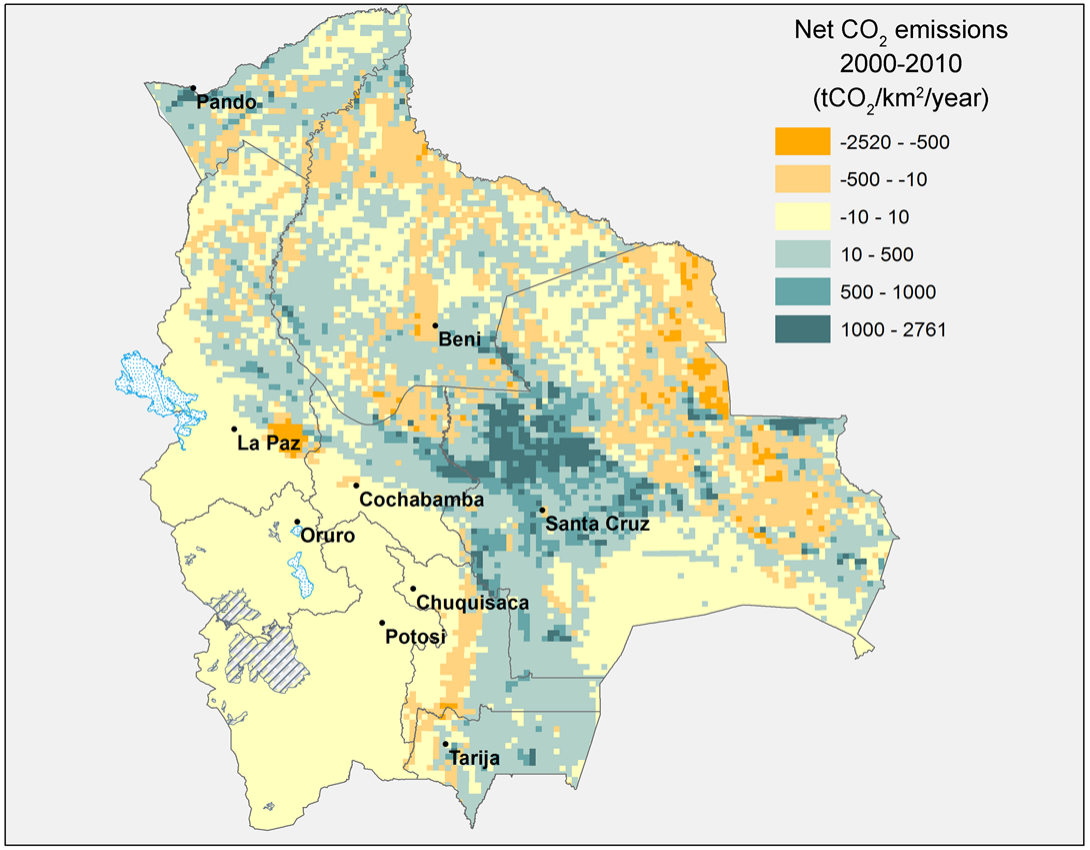
Annual net CO_2_ emissions from land use change, 2000–2010 (tCO_2_/km^2^/year). Source: Authors’ elaboration.

The overall average figures presented in Table 1 disguise highly heterogeneous trends across the country. Figs 4 and 5 below map annual net CO_2_ emissions in each pixel for the 1990s and 2000s, respectively.

During the 1990–2000 period (Fig 4), the majority of pixels (75%) registered neutral net carbon emissions from land use change (neutral here defined as having net CO_2_ emissions within the range of -10 to 10 tCO_2_ per square kilometre), while carbon emitting pixels (22% of all) were primarily concentrated in the commercially important department (state) of Santa Cruz with a limited number scattered across the remaining territory.

By 2000–2010, displayed in Fig 5, fewer pixels (45%) registered neutral emissions, with a greater proportion of both net carbon-emitting (39%) and net carbon-absorbing pixels (17%). While the centre of emissions is still just north of Santa Cruz de la Sierra, the spatial pattern of emissions and absorptions has become considerably more complex, with carbon-emitting and carbon-absorbing pixels displaying a less concentrated pattern.

As suggested by the maps above, the department of Santa Cruz is by far the biggest net CO_2_ emitter, but despite its emissions having increased over time, its share of total national emissions from land use change have dropped from 86.5% during the period 1990–2000 to 69.0% during 2000–2010. The department of Pando is now the second biggest net CO_2_ emitter, responsible for 7.9% of total net emissions during 2000–2010, up from only 2.2% during 1990–2000. Close behind follows Beni, which was responsible for 7.6% of total net emissions during 2000–2010. In contrast, Oruro, La Paz and Potosi have close to zero net emissions from land use change during the 2000–2010 period (see Table 2).

**Table 2:** Average annual net CO_2_ emissions from land use change, by department.

Department	Average annual net CO_2_ emissions from land use change (tCO_2_/year)		Share of national emissions from land use change	
	1990–2000	2000–2010	1990–2000	2000–2010
Chuquisaca	530,278	2,165,248	0.8%	2.3%
La Paz	622,517	1,438,699	1.0%	1.6%
Cochabamba	1,589,242	6,292,999	2.4%	6.8%
Oruro	0	0	0.0%	0.0%
Potosi	32	-23,789	0.0%	0.0%
Tarija	839,638	4,490,461	1.3%	4.8%
Santa Cruz	56,588,479	63,861,996	86.5%	69.0%
Beni	3,798,380	7,025,883	5.8%	7.6%
Pando	1,417,301	7,357,959	2.2%	7.9%
**Total**	**65,385,868**	**92,609,456**	**100.0%**	**100.0%**

Source: Authors’ estimations.

Land use changes and their associated carbon emissions are of course closely related to both demographics and economic activity. Table 3 presents estimated net CO_2_ emissions both per person and per unit of GDP produced in each department.

**Table 3:** Average annual net CO_2_ emissions from land use change, per capita and per unit of GDP, by department.

Department	Average per capita CO_2_ emissions (tCO_2_/person/year)		Average CO_2_ emissions per unit of GDP (kg/1990-Bs.)	
	1990–2000	2000–2010	1990–2000	2000–2010
Chuquisaca	1.1	3.9	0.5	1.7
La Paz	0.3	0.6	0.1	0.2
Cochabamba	1.3	4.0	0.5	1.4
Oruro	0.0	0.0	0.0	0.0
Potosi	0.0	0.0	0.0	-0.0
Tarija	2.6	10.6	0.9	1.9
Santa Cruz	36.3	28.5	10.7	8.0
Beni	12.6	18.4	5.5	7.3
Pando	33.4	106.9	9.6	29.4
**Bolivia**	**9.4**	**10.4**	**3.5**	**3.5**

Source: Authors’ estimations.

*Note*: GDP is measured in real (inflation-adjusted) Bolivianos of 1990 (1990-Bs.) so as to be comparable over time.

Santa Cruz, the agricultural centre of the country, used to be the biggest net CO_2_ emitter both per person and per unit of GDP, but has improved these indicators over time, while all other departments have seen deteriorations (except Oruro and Potosi which remain neutral in terms of emissions from land use change). Net *per capita* CO_2_ emissions from land use change in Santa Cruz have fallen by 21% between the 1990s and the 2000s, while emissions per unit of GDP have fallen by 25% (see Table 3).

Pando, the country’s most remote department, on the other hand, has seen large deteriorations in both indicators, despite having experienced very rapid growth in both population and GDP. Pando is now the second biggest CO_2_ emitter, responsible for 7.9% of total emissions during 2000–2010, up from only 2.2% during 1990–2000, and both per capita emissions and emissions per unit of GDP have increased three-fold (see Table 3).

**Fig 6:**
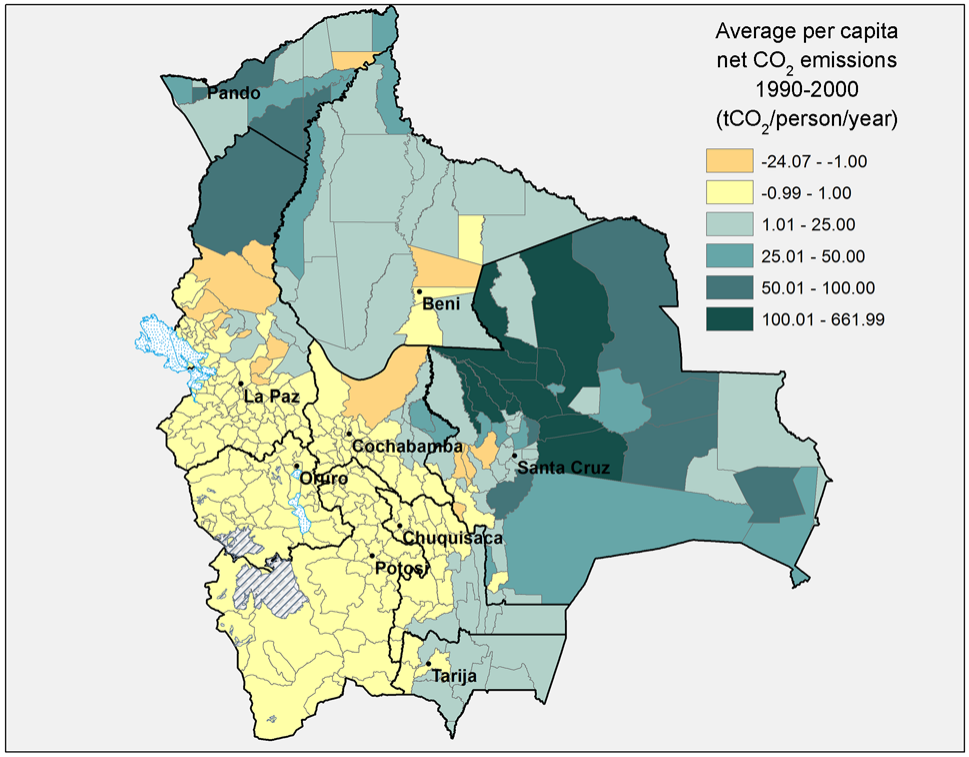
Average per capita CO_2_ emissions from land use change, 1990–2000, by municipality. Source: Authors’ elaboration.

**Fig 7:**
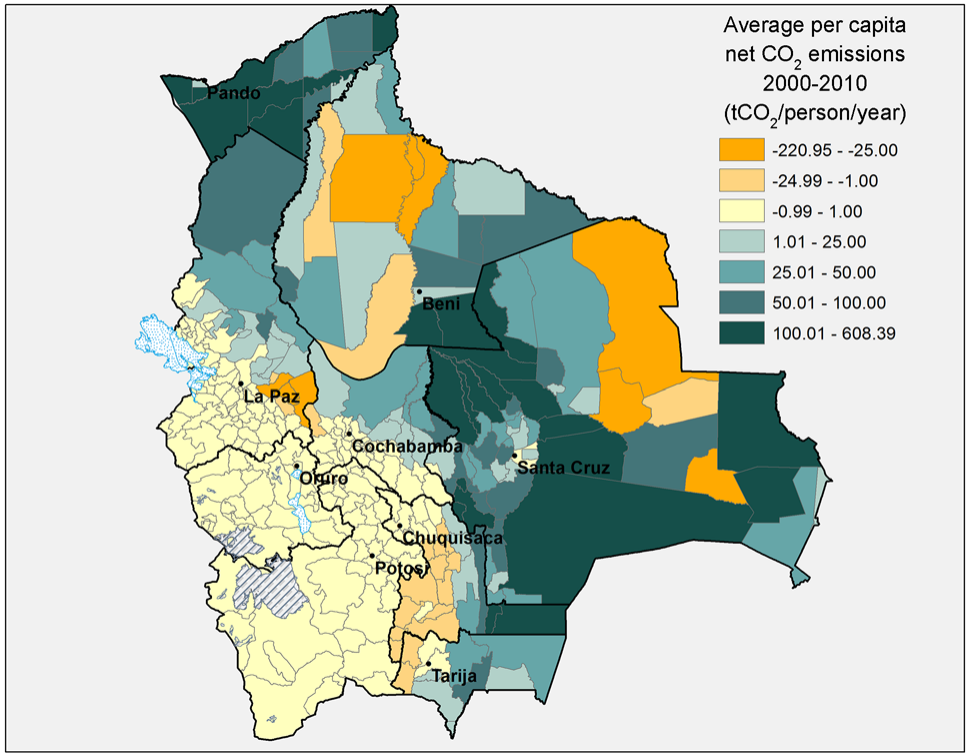
Average per capita CO_2_ emissions from land use change, 2000–2010, by municipality. Source: Authors’ elaboration.

Figs 6 and 7 below show average *per capita* CO_2_ emissions from land use change by municipality for the periods 1990–2000 and 2000–2010, respectively. From 1990–2000 only 15 out of 339 municipalities were significant net absorbers of CO_2_ (at least 1 tCO_2_ per person per year) due to land use change. From 2000–2010 this number had more than doubled to 31, and the magnitude of carbon absorption was also bigger. Notably, several municipalities changed from being big CO_2_ emitters in the 1990s to being significant CO_2_ sinks in the 2000s (especially in the departments of Santa Cruz, Beni, and the Yungas part of La Paz).

## Sensitivity Analysis

### Testing sensitivity to key assumptions

As outlined above, the Houghton et al. [17] bookkeeping model employed here required a number of assumptions and correspondingly raised a number of issues that could potentially influence the results. In particular, we test the degree to which our results vary according to: (a) the measure of above ground carbon used; (b) the proportion of agriculture assumed to be in pasture; (c) the degree to which below ground soil carbon might be released; (d) the assumed amount of carbon present in degraded forests; and (e) the degree of carbon emissions from static (unchanged) vegetation cover. The results of these exercises are presented in Tables 4 and 5.

**Table 4:** Sensitivity analysis of CO_2_ emissions from land use change in Bolivia, 2000–2010.

	Original baseline estimate (1)	Clearing less dense forest first (2)	20% pasture, 80% agriculture (3)	More aggressive agriculture (4)	Severely degraded forests (5)
Average annual CO_2_ emissions (tCO_2_/year)	92,609,456	82,445,942	90,244,388	97,339,593	99,928,918
Average annual per capita CO_2_ emissions (tCO_2_/person/year)	10.4	9.3	10.2	11.0	11.3

(1) See Table 1.

(2) We use the 25^th^ percentile of aboveground biomass instead of the 50^th^ percentile.

(3) Instead of 100% agriculture, we assume 20% pasture and 80% agriculture. No belowground emissions from pasture.

(4) We assume that 35% of belowground carbon is lost in 20 years instead of 25%.

(5) We assume that degraded forests have the carbon contents of 15 year old regrowth rather than 25 year old regrowth.

**Table 5:** Sensitivity analysis of CO_2_ emissions from lands that did not change use, 2000–2010.

	Central estimate (no emissions from lands with no use change) (1)	With emissions from desertification (2)	With absorption from mature forests (3)
Average annual CO_2_ emissions (tCO_2_/year)	92,609,456	94,286,643	7,185,963
Average annual per capita CO_2_ emissions (tCO_2_/person/year)	10.4	10.6	0.8

(1) See Table 1.

(2) We assume that 80% of non-forested land is losing 1% of soil organic carbon per year, and that 1.5% of this carbon is oxidized and emitted as CO_2_ to the atmosphere.

(3) We assume that aboveground biomass in mature forests increase by 4% per decade.

The first concern arises because even mature forests are not homogeneous (see Fig 1) and a number of studies (e.g. [11]; [21]; [22]) suggest that people tend to clear less dense forest first. If this is the case, then the average above ground carbon stored in those cleared forests may be lower than our assumed 50^th^ percentile figure that we used in the baseline model. We thus redo the analysis using instead the 25^th^ percentile. Column 2 of Table 4 shows that this assumption would reduce emissions by about 8% compared to the initial baseline estimate, reproduced in column 1.

The second experiment we do is to assume that not all forest cleared is for agriculture, but that 20% is used for pasture. Based on results reported by Guo and Gifford [24], we assume that when forest is converted to pasture, soil carbon remains intact, so for 20% of the cleared forest within each pixel we let below ground carbon remain intact rather than degrade by 25% as assumed in the original model. Reported in column 3 of Table 4, this change leads to a reduction in estimated emissions relative to baseline of about 3%.

The third potential issue is that changes in agricultural practices could release more or less carbon from the soils. For example, Schlesinger [28] reports that intensive tillage can cause soil to lose between 40 and 50% of its carbon. At the same time, conservation- or no-tillage practices have been credited for their ability to reduce greenhouse gas emissions [29], although the relationship between tillage and carbon emissions remains contested. For example, Lal [30] reports soil organic carbon sequestration ranges from 0.1 to 1.0 tC/ha per year from adopting no-tillage techniques, while Baker et al. [31] suggests such results could be due to insufficient sampling and finds no compelling evidence showing no-tillage practices favour carbon sequestration.

In Bolivia soybeans under 'conservation tillage' (e.g. no tillage) have increased from around 240,000 ha in 2000 to 726,207 ha in 2010 ([32]; [33]), currently constituting 82% of Bolivia’s soybean area. Thus if no-tillage fields are assumed to maintain most of soil carbon intact, the effect of this shift would be a reduction in total emissions similar to the experiment for pasture instead of agriculture. On the other hand, if the practices on the remaining agricultural lands are particularly aggressive, emissions from soils could be higher, as suggested by Schlesinger [28]. We thus adjust the proportion of below ground carbon that is assumed lost after 20 years of continuous intensive cultivation from 25% to 35% and find, as reported in column 4 of Table 4, that particularly aggressive agricultural practices would lead to a 5% increase in emissions compared to the baseline estimate.

In the final sensitivity experiment we assume that degraded forest is more severely degraded than in the baseline scenario, assuming that degraded forest has the same carbon contents as 15- year old, rather than 25-year old, regrowth, corresponding to an increase from approximately 15% loss of above ground carbon to a 59% loss. The result, presented in column 5 of Table 4, is that the much larger degradation would lead to 8% higher total CO_2_ emissions relative to baseline.

In addition to carbon released during land use change, total carbon emissions also includes carbon emissions from land whose vegetation cover (as viewed from a satellite) has not changed since 1990. For example, up to 41% of the Bolivian territory suffers from soil degradation/erosion/desertification [34], which may imply that soil organic carbon is being released to the atmosphere. In addition, there is evidence from permanent monitoring plots that biomass is increasing in mature, undisturbed forests in the Amazon (e.g. [35]; [36]). Since the areas of mature forests and degrading lands are orders of magnitude larger than the areas that have experienced changes in land use, even small changes in carbon stocks in these large areas can have a dramatic impact on the country’s net carbon emissions.

Unfortunately, despite their potentially great importance, no consensus yet exists on the extent of carbon emissions related to soil erosion [37]. Estimates range between 100% [38], to 50% [39], down to 0% [40], and even negative effects if there is significant deep burial of soil carbon at the deposition site (e.g. [41]; [42]). Wang et al. [43] shows that large amounts of carbon are transported from erosion sites to deposition sites during heavy rainfall events, but that only 1.5% of the transported carbon were oxidized into CO_2_, while the rest was stabilized and integrated into soils at the deposition site.

Given this uncertainty, we assume that on average 1% of soil organic carbon is lost annually from the 41% of the territory that is classified by Gardi et al. [34] as suffering from soil degradation. Adopting the experimental results of Wang et al. [43] we further assume that 1.5% of the carbon in eroded soils is oxidised into CO_2_, while the rest is deposited and integrated into soils in lower parts of the watersheds. As shown in column 2 of Table 5, taking soil erosion into account in this way only minimally changes our estimates of CO_2_ emissions.

On the other hand, Phillips et al. [35] suggests that mature forests across the basin added about 0.62 tC/ha/year to aboveground biomass during the last decades of the 20^th^ century. This corresponds to an increase of about 4% per decade. Column 3 of Table 5 presents the results of incorporating this insight. If we take into account this level of carbon absorption, net emissions during 2000–2010 drop by 92% compared to the central estimate that do not take into account carbon emissions or absorptions from lands with no recent use change.

### Comparing "Tier 3" and "Tier 1" estimates

Many estimates of carbon emissions rely on much more aggregated data that lack information on dynamic transitions and regrowth that we have incorporated into the bookkeeping "Tier 3" estimation methodology adopted in this study. It is thus an intriguing question to ask whether the extra information and dynamic structure we use imparts a significant improvement to the final results, and thus to what extent (and in which direction) more 'naive' estimates might be biased. To address this question we undertake a typical "Tier 1" calculation based on committed carbon emissions, simply multiplying average annual forest loss in Bolivia with average aboveground carbon contents in Bolivian forests (114 tC/ha), ignoring all the potential heterogeneity and dynamics, forest degradation, as well as any changes happening below ground. Average annual deforestation in the Tier 1 case is simply calculated as the area of mature forest by the beginning of the decade minus the area of mature forest by the end of the decade, divided by 10, not taking into account degraded or regenerating forest. The results, presented in Table 6, provide a reassuring answer; the 'naive' estimate of aggregate total CO_2_ emissions is only about 10% higher than our central baseline "Tier 3" estimate.

**Table 6:** "Tier 1" estimates of CO_2_ emissions from land use change in Bolivia, 1990–2000 and 2000–2010.

	1990–2000	2000–2010
Average annual forest loss (ha/year)	173,994	243,120
Average aboveground carbon density in Bolivian forests (tC/ha)	114	114
Average annual CO_2_ emissions (tCO_2_/year)	72,795,675	101,716,510
Mid-period population	6,987,201	8,873,150
Average annual per capita CO_2_ emissions (tCO_2_/person/year)	10.4	11.5

## Conclusions and Recommendations

This paper has analysed the net carbon emissions resulting from land use and land use changes in Bolivia during the periods 1990 to 2000 and 2000 to 2010. Using recently released highly detailed geolocated data on both land use changes over time and carbon contents in biomass and soils, we conduct an IPCC Tier 3 level estimation that take into account not only deforestation, but also forest degradation and forest regeneration. Our results indicate that net CO_2_ emissions from these processes amounted to almost one hundred million tons per year, corresponding to about 10 tons per person per year during the last two decades, estimates that are quite robust to reasonable changes in the underlying assumptions. If we allow for a potential long-run increase in the biomass of mature forests due, for example, to CO_2_ fertilisation [35], the estimates of net CO_2_ emissions from land use and land use change in Bolivia drop to close to zero.

At the sub-national level we see considerable heterogeneity in carbon emissions both between regions and over time. It is particularly interesting to note that the department of Santa Cruz, the agricultural powerhouse of Bolivia, has managed to lower both emissions per capita and emissions per person over time, despite rapid growth in the agricultural sector. This suggests that the agricultural sector in Santa Cruz is becoming more mature and efficient, which is in stark contrast to what is happening in the department of Pando in the far northern corner of Bolivia, where deforestation has increased substantially without concurrent improvements in GDP.

We also find that far simpler "Tier 1" estimates of carbon emission from land use change do very well, coming quite close to those from our more sophisticated "Tier 3" methodology. This is reassuring, and suggests that reliable carbon emissions estimates can be readily calculated as soon as rough deforestation estimates are available from global satellite image analysis. Thus it may not be necessary to invest in expensive and highly sophisticated carbon monitoring programs in order to know what is going on at the national level. Furthermore, it is likely that any gains in accuracy at the local level from such detailed monitoring would be offset by uncertainty about reference levels, problems of leakage, and possible absorption from mature forests. Ultimately our analysis suggests that in poorer countries resources may be best deployed in producing credible, official deforestation estimates annually.

## Supporting Information

S1 Supporting Information Forest types. (PDF)

S2 Supporting Information Land use transition matrices. (PDF)

S3 Supporting Information Above ground carbon contents. (PDF)

S4 Supporting Information Below ground carbon contents. (PDF)

S5 Supporting Information The age structure of fallow lands and above ground carbon regeneration parameters. (PDF)
