# Supplementary material for: Net Carbon Emissions from Deforestation in Bolivia during 1990-2000 and 2000-2010: Results from a Carbon Bookkeeping Model
Source: PLoS One. 2016 Mar 18;11(3):e0151241. doi: 10.1371/journal.pone.0151241 (PMC4798530; doi:10.1371/journal.pone.0151241)
Supplement: S1 Supporting Information — (PDF) [file pone.0151241.s001.pdf]

S1 Supporting Information: Forest types

In order to classify the different types of forests present in Bolivia, we use the official 2013 Forest Map published in MMAyA-OTCA [1]. This map divides all Bolivian forests into nine categories, adding up to a total forested area of 51,407,000 hectares in 2013 (see Table A).

Table A: Forest extension in Bolivia, 2013, by forest type

	**Forest extension**	
**Forest type**	**(ha)**	**(%)**
1. Amazon forest	19,402,388	37.7
2. Chaco forest	9,098,162	17.7
3. Chiquitano forest	8,645,849	16.8
4. Yungas forest	6,565,994	12.8
5. Tucumano forest	3,322,885	6.5
6. Flooded forest	3,047,598	5.9
7. Pantanal forest	1,147,401	2.2
8. Dry inter-Andean forest	172,227	0.3
9. Andean forest	4,496	0.01
**Total forest, 2013**	**51,407,000**	**100**

Source: MMAyA-OTCA [1].

References

1. Ministerio de Medio Ambiente y Agua, Sala de Observación Bolivia de la Organización del Tratado de Cooperación Amazónica. Memoria Técnica Mapa de Bosque 2013. Ministerio de Medio Ambiente y Agua; 2015.
