# Supplementary material for: Net Carbon Emissions from Deforestation in Bolivia during 1990-2000 and 2000-2010: Results from a Carbon Bookkeeping Model
Source: PLoS One. 2016 Mar 18;11(3):e0151241. doi: 10.1371/journal.pone.0151241 (PMC4798530; doi:10.1371/journal.pone.0151241)
Supplement: S2 Supporting Information — (PDF) [file pone.0151241.s002.pdf]

S2 Supporting Information: Land use transition matrices

Land use transition matrices were calculated for each pixel based on the data set produced by SERNAP [1], which analysed satellite images from NASA Geocover for 1990 and 2000; and from Landsat 5 TM for 2010 in order to determine land use and land use changes between these three periods. The majority of images were taken from the dry season (May to July) in order to obtain cloud-free images, but the exact dates vary from image to image and from year to year. For the initial processing, the Iterative Self-Organising Data Analysis Technique (ISODATA) was used to group the pixels according to the average value of each cluster into 150 classes. Then the ERDAS Grouping Tool was used to classify the 150 classes into 16 predetermined coverage classes such as types of forest, deforested areas, regenerating vegetation, water, cleared vegetation, and urban areas. Subsequently, a manual reclassification was applied for each pixel that had more than one class in order to determine the dominant class. Finally, all images were digitally united into a map of the whole territory, with a minimum mapping unit of 0.3 hectares. In the second stage, maps of land cover change induced by humans in forest areas were obtained by crossing vegetation cover maps for 1990, 2000, and 2010 using the Union Matrix tool from ERDAS.

Table B shows the aggregate land use transitions observed in Bolivia during the periods 1990-2000 and 2000-2010. This kind of matrices were calculated for each of our 11,150 pixels of 10 by 10 km.

Table B: Land use and land use change matrices for Bolivia, 1990-2000 and 2000-2010

(I) 1990-2000 (ha)

1990-2000	Forest 2000	Agro 2000	Regrowth 2000	Other 2000	Totals 1990
**Forest 1990**	55,002,123	1,439,908	229,662	88,331	56,760,024
**Agro 1990**	2,067	1,756,363	120,472	362	1,879,264
**Regrowth 1990**	493	144,756	513,396	1,069	659,714
**Other 1990**	13,405	5,629	1,362	48,706,765	48,695,313
**Totals 2000**	55,018,088	3,346,656	864,892	48,764,679	107,994,315

(II) 2000-2010 (ha)

2000-2010	Forest 2010	Agro 2010	Regrowth 2010	Other 2010	Totals 2000
**Forest 2000**	50,888,608	2,053,495	801,309	1,274,596	55,018,088
**Agro 2000**	241,390	2,675,745	339,528	89,993	3,346,656
**Regrowth 2000**	126,261	438,172	268,285	32,173	864,891
**Other 2000**	1,326,803	902	71,598	47,365,377	48,764,680
**Totals 2010**	52,583,062	5,168,314	1,480,800	48,762,139	107,994,315

Source: SERNAP [1].

References

1. Servicio Nacional de Áreas Protegidas. Deforestación y regeneración de bosques en Bolivia y en sus Áreas Protegidas Nacionales para los periodos 1990-2000 y 2000-2010. Servicio Nacional de Áreas Protegidas, Museo de Historia Natural Noel Kempff Mercado, Conservación Internacional – Bolivia, editors. La Paz: SERNAP; 2013.
