# Supplementary material for: Net Carbon Emissions from Deforestation in Bolivia during 1990-2000 and 2000-2010: Results from a Carbon Bookkeeping Model
Source: PLoS One. 2016 Mar 18;11(3):e0151241. doi: 10.1371/journal.pone.0151241 (PMC4798530; doi:10.1371/journal.pone.0151241)
Supplement: S3 Supporting Information — (PDF) [file pone.0151241.s003.pdf]

S3 Supporting Information: Above ground carbon contents

Roughly half of living biomass is carbon, so tons of biomass can be converted into tons of carbon by dividing by 2, as suggested by IPCC [1]. Applying this calculation to the median aboveground biomass contents from Fig 1 in the paper, we obtain a central estimate for aboveground carbon contents in intact forests as shown in Table C. Average aboveground carbon contents (weighted by the forest shares published in MMAyA-OTCA [2]) is 114 tC/ha.

Table C: Aboveground carbon contents for different types of intact forest

	**Aboveground carbon contents (tC/ha)**	
**Forest type**	**50th percentile**	**25th percentile**
1. Amazon forest	142	125
2. Chaco forest	64	59
3. Chiquitano forest	105	94
4. Yungas forest	142	130
5. Tucumano forest	92	76
6. Flooded forest	99	81
7. Pantanal forest	81	72
8. Dry inter-Andean forest	73	63
9. Andean forest	14	2
**Weighted average**	**114**	

Source: Authors' elaboration based on aboveground biomass measured by Baccini et al. [3].

The median values correspond quite well with IPCC's default values which are 150 tC/ha for tropical rain forests, 105 tC/ha for tropical dry forests, and 40 tC/ha for tropical scrubland in South America [1].

References

1. Intergovernmental Panel on Climate Change [IPCC]. Intergovernmental Panel on Climate Change Guidelines for National Greenhouse Gas Inventories. Chapter 4: Forest Lands. Japan: National Greenhouse Gas Inventories Programme; 2006. p 4.1-4.83.
2. Ministerio de Medio Ambiente y Agua, Sala de Observación Bolivia de la Organización del Tratado de Cooperación Amazónica. Memoria Técnica Mapa de Bosque 2013. Ministerio de Medio Ambiente y Agua; 2015.
3. Baccini A, Goetz SJ, Walker WS, Laporte NT, Sun M, Sulla-Menashe D, et al. Estimated carbon dioxide emissions from tropical deforestation improved by carbon-density maps. Nat Clim Chang. 2012; 2: 182–185.
