# Supplementary material for: Net Carbon Emissions from Deforestation in Bolivia during 1990-2000 and 2000-2010: Results from a Carbon Bookkeeping Model
Source: PLoS One. 2016 Mar 18;11(3):e0151241. doi: 10.1371/journal.pone.0151241 (PMC4798530; doi:10.1371/journal.pone.0151241)
Supplement: S4 Supporting Information — (PDF) [file pone.0151241.s004.pdf]

S4 Supporting Information: Belowground carbon contents

Apart from the carbon contained in the biomass aboveground, there are also large quantities of carbon stored belowground (in soils and live roots). These two carbon pools, identified by IPCC [1], are also affected by land use changes, although more slowly. To calculate soil carbon contents, we use the World Harmonized Soil Data Base [2] and assign the carbon contents of the dominant soil within each pixel. All of the organic carbon observations were extracted for both topsoil and subsoil for the dominant soil groups and units on the territory, except for Leptosols, the only soil group that doesn't have information for subsoil. This soil type is located mostly on the Andean, Dry Inter-Andean and Tucumano Forests in the southern part of the country.

No maps of carbon contents in roots exist for Bolivia, nor are there sufficient field samples available to create one, so we use IPCC's default ratios for belowground biomass to aboveground biomass for each forest type. Table D shows the parameters used.

Table D: Average carbon contents in roots, by forest type

Type of Forest	Ratio of below ground to above ground biomass (IPCC, 2006)	Carbon contents in roots (tC/ha)
1. Amazon forest	0.37	52
2. Chaco forest	0.56	36
3. Chiquitano forest	0.28	29
4. Yungas forest	0.24	34
5. Tucumano forest	0.37	34
6. Flooded forest	0.24	24
7. Pantanal forest	0.27	22
8. Dry inter-Andean forest	0.27	20
9. Andean forest	0.28	4

Source: Authors' elaboration based on IPCC [1] default values for ratios of belowground to aboveground biomass.

References

1. Intergovernmental Panel on Climate Change [IPCC]. Intergovernmental Panel on Climate Change Guidelines for National Greenhouse Gas Inventories. Chapter 4: Forest Lands. Japan: National Greenhouse Gas Inventories Programme; 2006. p 4.1-4.83.
2. Food and Agriculture Organization of the United Nations, International Institute for Applied Systems Analysis, International Soil Reference and Information Centre, Institute of Soil Science- Chinese Academy of Sciences, Joint Research Centre of the European Commission. Harmonized World Database (Version 1.2). 2013. Database: FAO. [Internet]. Accessed: http://www.fao.org/soils-portal/soil-survey/soil-maps-and-databases/harmonized-world-soil-database-v12/en/.
