# Supplementary material for: Net Carbon Emissions from Deforestation in Bolivia during 1990-2000 and 2000-2010: Results from a Carbon Bookkeeping Model
Source: PLoS One. 2016 Mar 18;11(3):e0151241. doi: 10.1371/journal.pone.0151241 (PMC4798530; doi:10.1371/journal.pone.0151241)
Supplement: S5 Supporting Information — (PDF) [file pone.0151241.s005.pdf]

S5 Supporting Information: The age structure of fallow lands and above ground carbon regeneration parameters

Our data set from SERNAP [1] provides a wall-to-wall data set of vegetation cover for the years 1990, 2000, and 2010. In order to fully describe the land use history of each plot in the country, however, a few assumptions are necessary regarding the first period of observation. Specifically, for 1990 we assume that the regrowth observed is only 5 years old and that before that, it had been used for agriculture for only 5 years. These assumptions are reasonable because significant deforestation in Bolivia only started in the early 1980s, when the collapse in the mining sector created massive unemployment and the Government tried to alleviate some of the social tensions by giving the affected workers free land in the forested Bolivian lowlands [2].

In subsequent periods we can explicitly track all land use changes. Thus, if a plot that showed 5-year regeneration in 1990 is still regenerating in 2000, then, by logic, that regenerating vegetation must be 15 years old in 2000. The oldest regenerating forest we can observe in the data set is 25 years old in 2010, and it could have been used for agriculture only for 5 years before that. Likewise, the oldest agricultural land we can observe is 25 years old in 2010. This means, for example, that soil carbon in this old regrowth has had sufficient time to fully recover to its original level.

Table E shows all the parameters that were used to calibrate the aboveground carbon regeneration curves for different vegetation types.

Table E: Maximum aboveground carbon contents, years to complete regeneration, and logistic regeneration parameters

Forest type	Maximum aboveground carbon contents (tC/ha)	Years to complete regeneration	α	β
1. Amazon forest	142	40	3.3105	0.1976
2. Chaco forest	64	35	2.4680	0.2018
3. Chiquitano forest	105	40	2.9957	0.1898
4. Yungas forest	142	40	3.3105	0.1976
5. Tucumano forest	92	35	2.8565	0.2129
6. Flooded forest	99	40	2.9339	0.1882
7. Pantanal forest	81	40	2.7213	0.1829
8. Dry inter-andean forest	73	35	2.6101	0.2059
9. Andean forest	14	35	0.5878	0.0771

Source: Authors' elaboration.

References

1. Servicio Nacional de Áreas Protegidas. Deforestación y regeneración de bosques en Bolivia y en sus Áreas Protegidas Nacionales para los periodos 1990-2000 y 2000-2010. Servicio Nacional de Áreas Protegidas, Museo de Historia Natural Noel Kempff Mercado, Conservación Internacional – Bolivia, editors. La Paz: SERNAP; 2013.
2. Klein HS. A Concise History of Bolivia. Cambridge: Cambridge University Press; 2003.
